# Health‐Related Quality of Life in The Chinese Cultural Context: A Scoping Review Protocol

**DOI:** 10.1002/hsr2.73322

**Published:** 2026-09-29

**Authors:** Shiwen Liu, Helen Rawson, Victoria Team

**Affiliations:** ^1^ Monash Nursing and Midwifery Monash University Melbourne Victoria Australia

**Keywords:** Chinese cultural context, Health‐related quality of life, quality of life, scoping review

## Abstract

**Background and Aims:**

While numerous measures of health‐related quality of life have been developed, most are grounded in Western perspectives and may not fully capture culturally specific values and beliefs. In Chinese culture, Confucianism, Taoism, and Buddhism shape people's perceptions of well‐being, health, and quality of life. These perceptions include some unique dimensions such as emotion control, weather adaptation, social adaptation, spirit, and complexion. Understanding these culturally embedded perspectives is essential for developing valid HRQoL measures, guiding patient‐centred care, and informing health policy. The aim of this protocol is to outline the process for a scoping review that will synthesise evidence on how quality of life and health‐related quality of life are understood among people from Chinese backgrounds.

**Methods:**

Studies published in English from database inception to 2 December 2025 will be searched in databases including Ovid Medline, Ovid Emcare, APA PsycINFO, CINAHL EBSCO, and Scopus. Qualitative or mixed‐methods studies with distinct qualitative components that report understandings of health‐related quality of life among adults from Chinese backgrounds will be included. The quality of included studies will be appraised using the Mixed Methods Appraisal Tool. A theory‐informed approach will be applied to data synthesis. Findings will be presented descriptively through tables and themes.

**Results:**

The findings of this scoping review will highlight variations within this cultural context, which may inform future efforts to enhance the cultural relevance of health‐related quality of life frameworks.

**Conclusion:**

In this protocol, we outline a transparent review process for a scoping review that will synthesise existing evidence on how quality of life and health‐related quality of life are perceived among people from Chinese backgrounds.

**Protocol Registration:**

This scoping review protocol has been deposited in the Open Science Framework and can be accessed at https://doi.org/10.17605/OSF.IO/GFC2P.

## 1 Introduction

Health‐related quality of life (HRQoL) has become an important concept that is widely discussed across health‐related disciplines. It reflects how individuals perceive and experience their own health, encompassing physical, psychological, and social dimensions [1]. As a multi‐dimensional concept, HRQoL is commonly used to assess how health status influences individuals' quality of life (QoL), as well as to evaluate medical treatments, guide clinical decision‐making, assess healthcare systems, monitor patient outcomes, and inform public health policies [2, 3]. HRQoL also offers critical insights into how diseases and medical interventions affect individuals' overall well‐being [4].

The term QoL is often used interchangeably with HRQoL in the literature, although the two terms are distinct [1]. QoL is a broader concept that captures individuals' overall life satisfaction and well‐being across multiple domains, whereas HRQoL specifically emphasises health‐related aspects of life, and is associated with the impact of disease and its treatments on individuals' QoL. [5] Both concepts are aimed at capturing the subjective perception of how health conditions and their treatments affect individuals' lives over time [1, 6].

As a multi‐dimensional construct, HRQoL is influenced by many factors, such as demographic characteristics, socioeconomic conditions, cultural background, and healthcare access [7]. Health is a culture‐rooted concept [8]. There are significant cultural differences in how health is defined, perceived and measured between Eastern and Western cultures [9]. In the Chinese cultural context, HRQoL exclusively includes some unique dimensions such as emotion control, weather adaptation, social adaptation, spirit, and complexion. These dimensions derive from Chinese idioms and traditional Chinese knowledge [9]. In addition, philosophical traditions such as Confucianism, Taoism, and Buddhism, together with collectivist values, play an important role in shaping how individuals perceive well‐being, health, and QoL [10]. In recent years, there has been increasing acknowledgment in China of the importance of introducing HRQoL into health management and research [11]. Although validated instruments such as the EQ‐5D, SF‐36, and WHOQOL‐100 have been adapted and applied in Chinese settings, they were originally developed in a Western context and may not fully reflect culturally specific understandings of health [9, 12]. A recent systematic review that compared Chinese‐developed HRQoL measures with Western‐developed instruments concluded that the latter may have limited applicability in non‐Western settings because they often do not capture culturally specific domains of well‐being [9].

Since there may be a mismatch or gap in understanding of HRQoL between the Chinese population and people from other cultural backgrounds, a scoping review will be conducted to examine how QoL and HRQoL are understood within the Chinese cultural context. This review will provide insights into culturally specific domains and interpretations of HRQoL within the Chinese cultural context, which can guide the subsequent analysis of empirical studies related to HRQoL among people from Chinese backgrounds with specific diseases. It will also help identify whether current HRQoL frameworks adequately capture cultural perspectives and, where possible, inform updates to HRQoL conceptual frameworks to ensure greater cultural relevance.

## 2 Methods

In this protocol for a scoping review, we present a transparent review process, reporting every step in accordance with the six‐stage scoping review framework described by Arksey and O'Malley [13] and further revised by Levac et al. [14]. The scoping review will be reported according to the Preferred Reporting Items for Systematic reviews and Meta‐Analyses extension for Scoping Reviews (PRISMA‐ScR) Checklist [15]. This review protocol was registered in the Open Science Framework (https://doi.org/10.17605/OSF.IO/GFC2P).

### 2.1 Stage One: Identifying the Research Question

In this scoping review, our objective is to systematically investigate and map the existing literature in order to address the following research question:

*How do Chinese people perceive HRQoL within their cultural context?*

### 2.2 Stage Two: Identifying Relevant Studies

As guided by Arksey and O'Malley's [13] framework, we will conduct a comprehensive search to identify eligible studies. A research librarian was consulted to support the development of the search strategy. An initial search of Ovid Medline was undertaken to identify keywords and subject headings from each concept related to the research question. The search strategies (Appendix 1) incorporate all keywords and index terms, which were adapted to each electronic database using phrases in quotations, truncations, proximity operator, Boolean operators and wildcards.

The following electronic databases will be searched from inception to 2 December 2025, including Ovid Medline, Ovid Emcare, Ovid PsycINFO, CINAHL EBSCO, and Scopus. Grey literature databases will not be searched in this review due to the PhD course time constraints. However, the reference list of all included articles will be screened for relevant articles.

### 2.3 Stage Three: Study Selection

The development of the inclusion and exclusion criteria for this scoping review was guided by the University of Melbourne Library guide (https://unimelb.libguides.com/sysrev/inclusion-exclusion-criteria). These criteria were developed around the key parameters of population, concept, context, study design, publication type, language, and geographic location of study, and were discussed in detail to ensure a transparent and replicable study selection process (Table 1).

**Table 1:** Inclusion and exclusion criteria.

	Inclusion criteria	Exclusion criteria
Population	Studies involving Adults aged 18 years and older from Chinese backgrounds, including those living in mainland China, Hong Kong, Macau, Taiwan, as well as those from Chinese backgrounds residing in other countries.	Studies involving only participants younger than 18 years. Studies involving only participants not from Chinese backgrounds.
Concept	Studies that explore perceptions or understandings of HRQoL or QoL among people from Chinese backgrounds. Studies identifying domains of generic HRQoL or QoL measures developed within the Chinese cultural context.	Disease‐specific QoL studies, or studies that do not explore HRQoL or QoL perceptions or understandings.
Context	Studies conducted in any setting that examine QoL or HRQoL within a Chinese cultural context	Studies not conducted in a Chinese cultural context
Study design	Qualitative studies. Mixed‐methods studies with a distinct qualitative component.	Experimental, quasi‐experimental, and observational studies. Studies focused only on translation, adaptation, or measurement properties of HRQoL or QoL instruments.
Publication type	Primary research studies	Reviews, abstract‐only studies, letters to the editor, commentaries, perspectives, and conference abstracts.
Language	Studies published in English.	Studies published in languages other than English
Geographic location of study	Studies from any geographic location, including those conducted in Chinese‐majority regions and in countries where Chinese populations reside	No exclusions based on geographic location

#### 2.3.1 Population

Studies involving participants aged 18 years and older from Chinese backgrounds will be included. These inclusion criteria encompass individuals living in mainland China, as well as Hong Kong, Macau, and Taiwan, and those from Chinese backgrounds residing in other countries. Studies will be excluded if involving only participants younger than 18 years old or only participants not from Chinese backgrounds.

#### 2.3.2 Concept

This scoping review will include studies investigating either HRQoL or QoL, as these terms are often used interchangeably in the literature. Qualitative studies that explore the understanding and perceptions of HRQoL among people from Chinese backgrounds, as well as qualitative studies aimed at identifying the domains of generic HRQoL measures developed within the Chinese cultural context, will be included. However, studies exploring disease‐specific QoL will be excluded because their findings are largely shaped by people's perspectives on and experiences of disease‐related symptoms and treatment effects. Including studies on disease‐specific QoL could obscure the cultural perspectives on general HRQoL that this review seeks to address.

#### 2.3.3 Context

Studies conducted in any setting, including urban or rural settings, healthcare settings, community settings, aged care settings, or other relevant environments, will be eligible for inclusion, as long as they explore HRQoL or QoL within a Chinese cultural context. Therefore, studies that do not examine HRQoL or QoL within a Chinese cultural context will be excluded.

#### 2.3.4 Study Design

In this scoping review, we will include qualitative studies that focus on the understanding of HRQoL among participants from Chinese backgrounds, such as phenomenology, grounded theory, ethnography, and qualitative description studies. In addition, mixed‐methods studies will be included if they conduct a distinct qualitative component that addresses the review objective.

Experimental, quasi‐experimental study designs, observational studies, and studies merely reporting the cultural adaptation, translation, or measurement properties of HRQoL or QoL measures will be excluded as they do not provide insight into participants' cultural perceptions and lived experiences of HRQoL.

#### 2.3.5 Publication Type

Primary research studies with full text will be included. Publications such as reviews, abstract‐only studies, letters to the editor, commentaries, perspectives, and conference abstracts will be excluded, as they do not provide primary qualitative data relevant to the objectives of this scoping review.

#### 2.3.6 Language

The review will be restricted to publications in English, reflecting the language proficiency of the review team.

#### 2.3.7 Geographic Location of Study

There will be no restrictions on the geographic location of study. Studies will not be limited to those conducted in mainland China, as studies involving people from Chinese backgrounds in other regions or countries will also be included.

#### 2.3.8 The Selection Process

The citations from all databases will be imported into Covidence [16] to identify and remove duplicates, and initiate the title and abstract screening and full‐text review. Two review authors will independently screen the titles and abstracts of all retrieved studies and select potentially eligible studies based on the eligibility criteria for full‐text review. EndNote 20 will be used to obtain the full text of the selected studies. If EndNote 20 is unable to retrieve the full texts of the selected studies, one review author will attempt to locate them through online searching. Following title and abstract screening, the two review authors will independently review the full texts and assess them against the eligibility criteria. Reasons for exclusion will be recorded in Covidence. Any discrepancies arising during the screening process will be resolved through discussions or by consultation with a third review author. The number of studies retrieved from each database, along with the number of studies included and excluded at each stage, and the reasons for exclusion, will be presented in a PRISMA flow diagram [17]. The PRISMA flow diagram template is shown in Figure 1.

**Figure 1:**
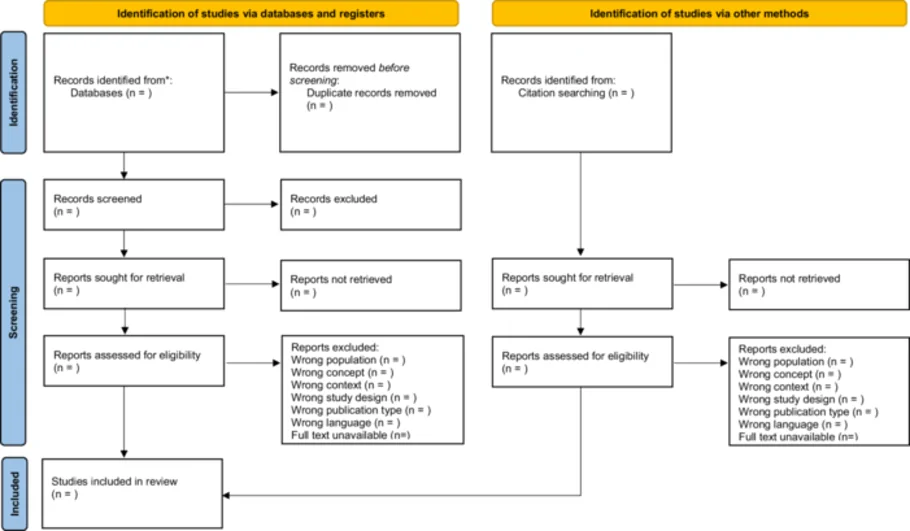
PRISMA 2020 flow [17].

### 2.4 Stage Four: Charting the Data

All data collected from the included studies will be aligned with the objective of this scoping review. A data extraction form developed by the review team will be used to collect relevant information, including author, year of publication, country, qualitative design, data collection and analysis method, study setting, sample size, participant characteristics including age and gender, education, the phenomena of interest, and Chinese cultural values/philosophical traditions. Data extraction will be conducted by one review author, and a second review author will independently check the extracted information for accuracy and completeness. Any discrepancy arising during this process will be resolved through discussion or by consulting with a third review author. During the data extraction process, further changes may be made to the data extraction form as needed. All modifications will be documented to ensure transparency and consistency across the review process.

### 2.5 Stage Five: Collating, Summarising and Reporting the Results

The included qualitative studies will be critically appraised by two independent review authors using the Mixed Methods Appraisal Tool (MMAT) [18]. Any discrepancies arising from this process will be resolved through discussion, or if necessary, by consulting with a third review author. The methodological quality of each study will be taken into account during data synthesis, and studies with lower quality ratings will be critically considered when interpreting the findings.

In this scoping review, data will be synthesised narratively to address the research question. Qualitative data will be subjected to a theory‐informed synthesis, guided by a working model of HRQoL developed by Mao et al [9] in their systematic review. This working model comprises four main domains: physical health, mental health, social health, and general health. It also includes 13 subdomains, which are physical symptoms, physical function, appearance, physical adaptation, negative affect, positive affect, cognitive function, mental adaptation, social relation, social function, social adaptation, general life satisfaction, and global health. In addition, this model contains sixty‐four concepts, and all domains and concepts were derived from either Western or Chinese HRQoL measures. Applying this conceptual model ensures that the synthesis of findings is grounded in a comprehensive and culturally inclusive framework, enabling an in‐depth interpretation of how HRQoL is understood among people from Chinese backgrounds.

Participant quotations, along with the interpretations provided by the study authors, will be extracted, charted, and coded by one review author, with NVivo 15 Software [19] used to support the coding process. Data will be collated by mapping extracted findings onto the existing domains, subdomains, and concepts within the working model, enabling the identification of key themes and subthemes. An inductive approach will be maintained to ensure openness to new concepts and subdomains that do not align with the current working model but may represent culturally relevant dimensions of HRQoL. A second review author will then independently verify all extracted quotations to ensure their alignment with the review objective and review the key themes. Any discrepancies arising during the data synthesis process will be resolved through discussion with all review authors.

### 2.6 Stage Six: Consultation

In this scoping review, we plan to conduct a consultation exercise to enhance the cultural relevance and validity of the findings. Individuals from Chinese cultural backgrounds from the authors' professional networks will be invited to provide feedback on the preliminary synthesis. Their perspectives will help determine whether the findings adequately reflect cultural understandings of HRQoL and will allow for identifying additional insights that are not captured in the published literature. Information obtained during this stage will be incorporated into the final analysis to strengthen the cultural applicability of the review outcomes.

## 3 Ethics and Dissemination

Since this scoping review will collect data from publicly accessible sources and the consultation component will be undertaken as a public consultation, ethical approval is not required. The findings of this scoping review will be submitted for publication in a peer‐reviewed journal, presented at relevant national or international conferences, or research seminars.

## 4 Conclusion

This protocol presents the introduction, rationale, and objective, and outlines a transparent process for conducting a scoping review that aims to examine how QoL and HRQoL are understood within the Chinese cultural context. This scoping review will map the available evidence on how individuals from Chinese backgrounds perceive HRQoL. This scoping review may be limited by the exclusion of unpublished studies and articles written in languages other than English, including Chinese‐language literature, which may contain relevant information on this topic. Despite these limitations, the findings are expected to provide valuable insights that inform future research aimed at improving the understanding of HRQoL among people from Chinese backgrounds. In addition, the findings will highlight variations within this cultural context, which may inform future efforts to enhance the cultural relevance of HRQoL frameworks, thereby supporting the future development or adaptation of HRQoL measures grounded in a more culturally inclusive conceptual foundation. The findings may also contribute to the work of health professionals, policymakers, and researchers in developing culturally sensitive approaches to improve HRQoL among Chinese populations affected by specific diseases or health conditions.

## Author Contributions

**Shiwen Liu:** conceptualization, methodology, investigation, data curation, writing – original draft, writing – review and editing. **Helen Rawson:** conceptualization, methodology, data curation, writing – review and editing, supervision. **Victoria Team:** conceptualization, methodology, investigation, data curation, writing – review and editing, supervision. All authors have read and approved the final version of the manuscript. Corresponding author (Shiwen Liu) has full access to all of the data in this study and takes complete responsibility for the integrity of the data and the accuracy of the data analysis.

Shiwen Liu is the recipient of the Australian Government Research Training Program (RTP) Scholarship for conducting a Ph.D. project, including the present work. The funder was not involved in the study design; data collection, analysis, or interpretation of data; the writing of this manuscript; or the decision to submit for publication. Open access publishing facilitated by Monash University, as part of the Wiley ‐ Monash University agreement via the Council of Australasian University Librarians.

## Conflicts of Interest

The authors declare no conflicts of interest.

## Transparency Statement

Shiwen Liu affirms that this manuscript is an honest, accurate, and transparent account of the study being reported; that no important aspects of the study have been omitted; and that any discrepancies from the study as planned.

## Supporting information


Supporting File

## Data Availability

Data sharing is not applicable to this article as this review protocol does not involve the collection or generation of new empirical data.
