# Supplementary material for: Health‐Related Quality of Life in The Chinese Cultural Context: A Scoping Review Protocol
Source: Health Sci Rep. 2026 Sep 29;9(10):e73322. doi: 10.1002/hsr2.73322 (PMC13624285; doi:10.1002/hsr2.73322)
Supplement: Supplementary file 1 [file HSR2-9-e73322-s001.docx]

# 1. Appendix

## Database-Specific Search Strategy

### Database: Ovid MEDLINE(R)

| **#** | **Query** | **Results from 02 Dec 2025** |
| --- | --- | --- |
| 1 | (China* or Chinese or Han or Hong Kong or Hongkong* or Taiwan* or Macau or Macao or "Chinese people" or "Chinese background*" or "Chinese cultur*" or "Chinese population" or "people in China" or "Chinese immigrant*" or "Chinese migrant*" or "overseas Chinese").mp. | 729,174 |
| 2 | exp "Quality of Life"/ | 314,664 |
| 3 | ("quality of life" or "QoL" or "health-related quality of life" or "HRQoL" or "HRQL" or "life qualit*").mp. | 462,258 |
| 4 | 2 or 3 | 463,008 |
| 5 | Attitude to Health/ | 85,682 |
| 6 | (("underst*" not "understat*") or percept* or "concept*" or "cultural belief*" or "belief*" or "value*" or "perspective*" or "cultural context" or "cultural influenc*" or meaning* or experienc* or "lived experience*").mp. | 6,453,436 |
| 7 | 5 or 6 | 6,494,927 |
| 8 | 1 and 4 and 7 | 7,624 |
| 9 | "Document Analysis"/ or focus groups/ or interviews as topic/ or narration/ or qualitative research/ or ((depth or face or group or guided or indepth or informal or semistructured or structured or unstructured) adj4 (discussion or discussions or interview or interviewed or interviews or questionnaire or questionnaires)).ti,ab,kf,kw. or (ethnographic or ethnography or (field adj1 work) or fieldwork or (focus adj1 (group or groups)) or (groups adj2 interviewed) or (key adj1 (informant or informants)) or (qualitative adj2 (research or studies or studies or synthesis))).ti,ab,kf,kw. | 374,051 |
| 10 | 8 and 9 | 828 |
| 11 | limit 10 to English language | 797 |

### Database: Ovid Emcare

| **#** | **Query** | **Results from 2 Dec 2025** |
| --- | --- | --- |
| 1 | exp Chinese/ | 37,282 |
| 2 | (China* or Chinese or Han or Hong Kong or Hongkong* or Taiwan* or Macau or Macao or "Chinese people" or "Chinese background*" or "Chinese cultur*" or "Chinese population" or "people in China" or "Chinese immigrant*" or "Chinese migrant*" or "overseas Chinese").mp. | 292,806 |
| 3 | 1 or 2 | 292,806 |
| 4 | exp "quality of life"/ | 281,340 |
| 5 | ("quality of life" or "QoL" or "health-related quality of life" or "HRQoL" or "HRQL" or "life qualit*").mp. | 330,208 |
| 6 | 4 or 5 | 330,208 |
| 7 | exp attitude to health/ | 27,954 |
| 8 | (("underst*" not "understat*") or percept* or "concept*" or "cultural belief*" or "belief*" or "value*" or "perspective*" or "cultural context" or "cultural influenc*" or meaning* or experienc* or "lived experience*").mp. | 2,772,494 |
| 9 | 7 or 8 | 2,784,693 |
| 10 | 3 and 6 and 9 | 5,849 |
| 11 | exp qualitative research/ | 115,417 |
| 12 | exp interview/ | 329,363 |
| 13 | (((depth or face or group or guided or indepth or informal or semistructured or structured or unstructured) adj4 (discussion or discussions or interview or interviewed or interviews or questionnaire or questionnaires)) or (ethnographic or ethnography or (field adj1 work) or fieldwork or (focus adj1 (group or groups)) or (groups adj2 interviewed) or (key adj1 (informant or informants)) or (qualitative adj2 (research or studies or studies or synthesis)))).ti,ab,kf,kw. | 292,352 |
| 14 | 11 or 12 or 13 | 466,199 |
| 15 | 10 and 14 | 892 |
| 16 | limit 15 to English language | 860 |

### Database: APA PsycInfo

| **#** | **Query** | **Results from 2 Dec 2025** |
| --- | --- | --- |
| 1 | (China* or Chinese or Han or Hong Kong or Hongkong* or Taiwan* or Macau or Macao or "Chinese people" or "Chinese background*" or "Chinese cultur*" or "Chinese population" or "people in China" or "Chinese immigrant*" or "Chinese migrant*" or "overseas Chinese").mp. | 138,835 |
| 2 | exp "quality of life"/ | 60,330 |
| 3 | ("quality of life" or "QoL" or "health-related quality of life" or "HRQoL" or "HRQL" or "life qualit*").mp. | 123,222 |
| 4 | 2 or 3 | 124,693 |
| 5 | exp health attitudes/ | 28,947 |
| 6 | (("underst*" not "understat*") or percept* or "concept*" or "cultural belief*" or "belief*" or "value*" or "perspective*" or "cultural context" or "cultural influenc*" or meaning* or experienc* or "lived experience*").mp. | 2,886,602 |
| 7 | 5 or 6 | 2,895,633 |
| 8 | 1 and 4 and 7 | 2,605 |
| 9 | focus group/ or focus group interview/ or grounded theory/ or interpretative phenomenological analysis/ or narrative analysis/ or qualitative measures/ or qualitative methods/ or semi-structured interview/ or thematic analysis/ or (focus group or interview or qualitative study).md. or ("Document Analysis" or focus groups or "interviews as topic" or narration or "qualitative research").mh. or ((depth or face or group or guided or indepth or informal or semistructured or structured or unstructured) adj4 (discussion or discussions or interview or interviewed or interviews or questionnaire or questionnaires)).ti,ab,id. or (ethnographic or ethnography or (field adj1 work) or fieldwork or (focus adj1 (group or groups)) or (groups adj2 interviewed) or (key adj1 (informant or informants)) or (qualitative adj2 (research or studies or studies or synthesis))).ti,ab,id. | 692,240 |
| 10 | 8 and 9 | 777 |
| 11 | limit 10 to English language | 759 |

### Database: CINAHL Complete

| S1 | MH "Chinese Persons" OR XB (China* OR Chinese OR Han OR Mandarin OR Cantonese OR Hong Kong OR Hongkong* OR Taiwan* OR Macau OR Macao OR Chinese people OR Chinese background* OR Chinese cultur* OR Chinese population OR people in China OR Chinese immigrant* OR Chinese migrant* OR overseas Chinese) |
| --- | --- |
| S2 | MH "Quality of Life" OR XB (quality of life OR QoL OR health-related quality of life OR HRQoL OR HRQL OR life qualit*) |
| S3 | MH "Attitude to Health" OR TX (underst* NOT understat* OR percept* OR concept* OR cultural belief* OR belief* OR value* OR perspective* OR cultural context OR cultural influenc* OR meaning* or experienc* OR lived experience*) |
| S4 | MH "Qualitative Studies" OR MH "Grounded theory" OR MH "Narratives" OR MH "Interviews+" OR MH "Audiorecording" OR MH "Focus Groups" OR MH "Discourse Analysis" OR MH "Content Analysis" OR MH "Ethnographic Research" OR MH "Ethnonursing Research" OR MH "Constant Comparative Method" OR MH "Qualitative Validity+" OR MH "Purposive Sample" OR MH "Observational Methods+" OR MH "Field Studies" OR MH "Theoretical Sample" OR MH Phenomenology OR MH "Phenomenological Research" OR MH "Life Experiences+" OR MH "Cluster Sample+" OR TI qualitative OR AB qualitative OR TI interview* OR AB interview* OR TI (theme* or thematic) OR AB (theme* or thematic) OR TI ("ethnological research") OR AB ("ethnological research") OR TI ethnonursing OR AB ethnonursing OR TI ethnograph* OR AB ethnograph* OR TI phenomenol* OR AB phenomenol* OR TI "focus group*" OR AB "focus group*" OR TI (grounded N1 (theor* OR analys?s OR research OR studies OR study)) OR AB (grounded N1 (theor* OR analys?s OR research OR studies OR study)) OR TI ("life stor*") OR AB ("life stor*") OR TI (emic OR etic OR hermeneutic* OR heuristic* OR semiotic) OR AB (emic OR etic OR hermeneutic* OR heuristic* OR semiotic) OR TI (data N1 saturat*) OR AB (data N1 saturat*) OR TI ("participant observ*") OR AB ("participant observ*") OR TI ("social construct*" OR postmodern* OR "post-structural*" OR poststructural* OR "post-modern*" OR feminis*) OR AB ("social construct*" OR postmodern* OR "post-structural*" OR poststructural* OR "post-modern*" OR feminis*) OR TI ("action research" OR "cooperative inquir*" OR "co-operative inquir*") OR AB ("action research" OR "cooperative inquir*" OR "co-operative inquir*") OR TI (humanistic OR existential OR experiential OR paradigm*) OR AB (humanistic OR existential OR experiential OR paradigm*) OR TI (field N1 (research OR study OR studies)) OR AB (field N1 (research OR study OR studies)) OR TI "human science" OR AB "human science" OR TI "biographical method" OR AB "biographical method" OR TI ("theoretical sampl*") OR AB ("theoretical sampl*") OR TI ("purpos* N4 sampl*") OR AB ("purpos* N4 sampl*") OR TI ("open-ended" OR narrative* OR textual OR texts OR "semi-structured") OR AB ("open-ended" OR narrative* OR textual OR texts OR "semi-structured") OR TI ("life world" OR "life-world" OR "conversation analys?s" OR "personal experience*" OR "theoretical saturation") OR AB ("life world" OR "life-world" OR "conversation analys?s" OR "personal experience*" OR "theoretical saturation") OR TI ((life OR lived) N1 experience*) OR AB ((life OR lived) N1 experience*) OR TI ("cluster sampl*") OR AB ("cluster sampl*") OR TI ("observational method*") OR AB ("observational method*") OR TI ("content analysis") OR AB ("content analysis") OR TI ((discurs* OR discourse*) N3 analys?s) OR AB ((discurs* OR discourse*) N3 analys?s) OR TI (constant N1 (comparison OR comparative)) OR AB (constant N1 (comparison OR comparative)) OR TI ("narrative analys?s") OR AB ("narrative analys?s") OR TI (heidegger* OR colaizzi* OR spiegelberg* OR merleau* OR husserl* OR foucault* OR ricoeur OR glaser*) OR AB (heidegger* OR colaizzi* OR spiegelberg* OR merleau* OR husserl* OR foucault* OR ricoeur OR glaser*) OR TI (van N1 manen*) OR AB (van N1 manen*) OR TI (van N1 kaam*) OR AB (van N1 kaam*) OR TI (Corbin* N2 strauss*) OR AB (Corbin* N2 strauss*) |
| S5 | S1 AND S2 AND S3 **Limiters** - English Language |

### Database: Scopus

(TITLE-ABS-KEY(( China* OR Chinese OR Han OR Hong Kong OR Hongkong* OR Taiwan* OR Macau OR Macao OR "Chinese people" OR "Chinese background*" OR "Chinese cultur*" OR "Chinese population" OR "people in China" OR "Chinese immigrant*" OR "Chinese migrant*" OR "overseas Chinese" )) AND TITLE-ABS-KEY(( "quality of life" OR "QoL" OR "health-related quality of life" OR "HRQoL" OR "HRQL" OR "life qualit*" )) AND TITLE-ABS-KEY(( ( "underst*" NOT "understat*" ) OR percept* OR "concept*" OR "cultural belief*" OR "belief*" OR "value*" OR "perspective*" OR "cultural context" OR "cultural influenc*" OR meaning* OR experienc* OR "lived experience*" )) AND TITLE-ABS-KEY(( "Empirical Research" OR interview OR "Interviews as Topic" OR "Personal Narratives" OR "Focus Groups" OR narration OR "Nursing Methodology Research" OR "Narrative Medicine" OR interview* OR qualitative OR theme* OR thematic OR "ethnological research" OR ethnograph* OR ethnomedicine OR ethnonursing OR phenomenol* OR "grounded theor*" OR "grounded study" OR "grounded studies" OR "grounded research" OR "grounded analysis" OR "grounded analyses" OR "life stor*" OR emic OR etic OR hermeneutic* OR heuristic* OR semiotic* OR "data saturat*" OR "participant observ*" OR "social construct*" OR postmodern* OR post-structural* OR "post structural*" OR poststructural* OR "post modern*" OR post-modern* OR feminis* OR "action research" OR "cooperative inquir*" OR "co operative inquir*" OR humanistic OR existential OR experiential OR paradigm* OR "field study" OR "field studies" OR "field research" OR "field work" OR "human science" OR "social science" OR "biographical method" OR "theoretical sampl*" OR ( purpos* W/3 sampl* ) OR ( focus W/3 group* ) OR open-ended OR narrative* OR textual OR texts OR "semi-structured" OR "life world*" OR "life-world*" OR "conversation analysis" OR "personal experience*" OR "theoretical saturation" OR "conversation analyses" OR "lived experience" OR "life experience*" OR "cluster sampl*" OR "observational method*" OR "content analysis" OR "constant comparative" OR "constant comparison" OR ( discourse* W/3 analysis ) OR ( discourse* W/3 analyses ) OR ( discurs* W/3 analysis ) OR ( discurs* W/3 analyses ) OR heidegger* OR colaizzi* OR spiegelberg* OR merleau* OR husserl* OR foucault* OR ricoeur OR glaser* OR "van manen*" OR "van kaam*" OR ( corbin* W/2 strauss* ) ))) AND ( LIMIT-TO ( LANGUAGE,"English" ) )
